# Current Updates on Molecular Diagnostic Assays Used for Detection of *Candida auris*: A Systematic Review

**DOI:** 10.3390/diagnostics15020140

**Published:** 2025-01-09

**Authors:** River Chun-Wai Wong, Alfred Lok-Hang Lee, Ingrid Yu-Ying Cheung, Viola Chi-Ying Chow, Margaret Ip, Christopher Koon-Chi Lai

**Affiliations:** 1 Department of Microbiology, Prince of Wales Hospital, Hospital Authority, Hong Kong SAR, China; llh849@ha.org.hk (A.L.-H.L.); cyy402@ha.org.hk (I.Y.-Y.C.); chowcyv@ha.org.hk (V.C.-Y.C.); 2 Department of Microbiology, Faculty of Medicine, The Chinese University of Hong Kong, Hong Kong SAR, China; margaretip@cuhk.edu.hk

**Keywords:** *Candida auris*, molecular diagnostics, sample pre-extraction treatment, nucleic acid extraction, laboratory-developed molecular assay, commercial molecular assay

## Abstract

**Background/Objectives**: *Candida auris* is an emerging multidrug-resistant pathogen with the potential to cause invasive fungal infections and healthcare-associated outbreaks. Currently, there is no systematic review explicitly focusing on the up-to-date molecular diagnostics of this pathogen to cover the entire process, including sample pre-extraction procedures, nucleic acid extraction, and DNA-based detection. Sample pre-treatment and extraction are the prerequisites before molecular testing and have implications on the downstream detection but have not been reviewed elsewhere. This review aims to summarize a comprehensive update in the past 5 years. **Methods**: A systematic review was conducted to search for articles published in the period between 1 January 2020 and 20 November 2024 from various databases, including PubMed, Google Scholar, and Web of Science. The findings were produced through narrative synthesis, with quantitative analysis conducted where applicable. **Results**: Starting from 1115 records, 28 studies that met the inclusion criteria were included in the analysis. This review summarized the key updates on three categories, including (i) sample pre-extraction procedures and nucleic acid extraction, including magnetic, bead-beating, mechanical, chemical, thermal, and column-based protocols; (ii) commercial molecular assays; and (iii) laboratory-developed tests (LDTs). For real-time PCR, commercial molecular assays and LDTs showed sensitivity (ranging from 94.9% to 100% and 44% to 100%, respectively) and specificity (ranging from 98.2% to 100% and 92% to 100%, respectively). **Conclusions**: Here, we describe a useful summary to enlighten readers from clinical microbiology laboratories on the nucleic acid extraction protocols and performance of various molecular diagnostic assays used for the detection of *C. auris*.

## 1. Introduction

*Candida auris* is an emerging multidrug-resistant pathogen with the potential to cause invasive fungal infections and healthcare-associated outbreaks. In 2022, the World Health Organization categorized this opportunistic organism into the critical priority group of the fungal priority pathogens list, which indicates the rising threat of *C. auris* to global public health [1]. It was first reported in 2009 in Tokyo, Japan, and rapidly disseminated worldwide in the past decade [2]. This multidrug-resistant pathogen is increasingly detected, becoming prevalent, and is considered endemic in many parts of Asia, as well as causing nosocomial outbreaks in the U.S. and many European countries (e.g., U.K., Italy, and Spain). [3]. Phylogenetic studies show that it can further differentiate into six distinct clades based on its molecular epidemiology and geographical locations (i.e., Clade I (South Asia), Clade II (East Asia), Clade III (South Africa), Clade IV (South America), Clade V (Iran)), and Clade VI (Singapore) [3,4,5,6,7,8,9,10,11,12,13,14,15,16,17,18,19]. Unlike other *Candida* species, *C. auris* is highly resistant to fluconazole, which limits its treatment options, while it also has moderate (40%) and low (2%) resistance rates to amphotericin B and echinocandins, respectively [1,10,19,20,21,22,23]. Among the six distinct clades, Clade I has the highest multidrug-resistant reported rates to azoles and amphotericin B, while Clade II is relatively susceptible to antifungal and disinfectants [4,5]. Clade III is mostly resistant to azoles but seldomly resistant to amphotericin B. Generally, echinocandins are considered as the first-line treatment against *C. auris*; however, a high resistance rate to this drug has been reported in Clade IV isolates [4,5,16,19,21,24,25]. Clade II mainly infects the ears, while other clades are associated with invasive infections and nosocomial outbreaks [19].

This formidable fungal pathogen has a propensity to colonize on the surface of human skin (e.g., external ear canal, axilla, groin, etc.), hands, nares, and the healthcare environment [4,17,19,24,26,27]. Given the widespread transmissibility of *C. auris*, it can spread between patients and contaminated items and form persistent biofilms in clinical environments, leading to healthcare-associated outbreaks [3,4,6,8,9,11,12,13,14,15,17,19,21,22,23,24,25,26,28,29,30,31]. This pathogen is difficult to eradicate once colonized and is associated with prolonged hospital outbreaks, while daily skin decolonization with 2% aqueous chlorhexidine wipes would be required for colonized patients [3,19,32]. *C. auris* can also cause life-threatening disease and severe invasive fungal infections (e.g., candidemia) and is associated with a high mortality rate in immunocompromised patients [3,7,9,11,13,14,15,19,22,23,25,29,31,33,34,35].

The rapid identification of *C. auris* in both symptomatic patients and asymptomatic carriers is crucial for effective implementation of infection control measurements and to prevent nosocomial transmission [4,10,12,16,21,22,24,26,29,33,35,36]. Laboratories are required to increase their diagnostic capacity to circumvent the spread of this fungal pathogen. In regions where *C. auris* is endemic, active surveillance screening is widely recommended on inpatient admission with known risk factors (e.g., recent hospitalization in endemic hospitals or nursing homes or close contact with a confirmed *C. auris* case) to identify colonization and infections [3,6,8,9,12,16,17,22,37]. The CDC recommends extensive screening on patients (i.e., a point prevalence survey) if ongoing nosocomial transmission has been identified [9,17,25,38]. Although culture-based methodologies are low-cost and considered as the gold standard, they are time-consuming and *C. auris* can also be misidentified by some phenotypic identification methods [5,10,16,21,22,23,25,31,34,39,40]. Alternatively, a molecular-based diagnostic method can be performed on patient samples directly, reduces the results’ turn-around time (TAT) significantly, and has good diagnostic sensitivity and specificity. It is particularly useful in outbreak containment [5,8,10,16,17,21,23,24,26,34,36].

Despite the widespread need for rapid detection of *C. auris* by molecular diagnostics, commercially available real-time PCR assays are still scarce in the market [28]. Currently, there are two US Food and Drug Administration (FDA)-approved molecular assays validated for testing on positive blood culture samples (i.e., GenMark ePlex Blood Culture Identification Fungal Pathogen (BCID-FP) and BioFire FilmArray BCID2 Panel); an FDA-approved assay for other specimen types in the molecular detection of *C. auris* is not yet available [7,26]. Clinical microbiology laboratories are therefore responsible for developing their own molecular protocols, such as nucleic extraction methods, real-time PCR, loop-mediated isothermal amplification (LAMP), and sequencing [15,24,26]. Commercially available assays and laboratory-developed tests (LDTs) are mainly targeting the genetic loci located in the specific regions of ribosomal DNA (rDNA) genes of *C. auris* (i.e., 18S rDNA, D1/D2 region of 28S rDNA, and internal transcribed spacers (*ITS1*/*ITS2*)) [8,11,17,24,30,31,34,36]. Species identification can also be performed by Sanger sequencing and/or whole genome sequencing (WGS) [11,16,17,30,41].

A review has been reported recently focusing on the outbreak evaluation of *C. auris* through molecular assays and antifungal stewardship [28]; however, such a review is based on a narrative approach, not a systematic review. Moreover, molecular diagnostics that are applied in routine screening would have a clinical impact on patient management, whereas it has not been addressed previously. To our knowledge, there is no systematic review explicitly focusing on the up-to-date molecular diagnostics of this pathogen and covering the sample pre-extraction procedures and nucleic acid extraction. To release the genomic DNA effectively, *C. auris* cells should be completely lysed for nucleic acid extraction prior to DNA-based detection. This article covers the sample pre-extraction procedures and nucleic acid extraction, including magnetic, bead-beating, mechanical, chemical, thermal, and column-based protocols. They are prerequisites before molecular testing and have implications on downstream DNA-based detection but have not been reviewed elsewhere. We aim to summarize a comprehensive update in the past 5 years (2020–2024) to provide valuable insights and discuss the relevant aspects to the readers from clinical microbiology laboratories.

## 2. Materials and Methods

### 2.1. Study Design

This systematic review was conducted under the 2020 Preferred Reporting Items for Systematic Review and Meta-Analyses (PRISMA) guidelines [42,43]. A comprehensive search was performed on 20 November 2024 for potentially eligible studies published in the period between 1 January 2020 and 20 November 2024 on various electronic databases with full text available. The databases covered in this review included PubMed, Google Scholar, and Web of Science.

### 2.2. Search Strategy

For the PubMed database, the search was performed through the Advanced Search Builder using the keyword term in the title/abstract for “*Candida auris*” and then combined with criteria terms, using AND terms, including (“molecular” [Title/Abstract] OR “diagnostic” [Title/Abstract] OR “diagnosis” [Title/Abstract] OR “screening” [Title/Abstract] OR “detection” [Title/Abstract] OR “PCR” [Title/Abstract] OR “identification” [Title/Abstract] OR “method” [Title/Abstract]).

For Google Scholar, the search was performed through the advanced search function using the following keywords under the criteria “with all of the words”—“*Candida auris*”—and with the following terms under the criteria “with at least one of the words”—“molecular”, “diagnostic”, “diagnosis”, “screening”, “detection”, “PCR”, “identification”, and “method”. Then, “anywhere in the article” was selected for “where my words occur”.

For Web of Science, the search was performed through the advanced search using the title with the following strings: “TI = (*Candida auris*)” AND “TI = (molecular) AND TI = (diagnostic) OR TI = (diagnosis) OR TI = (screening) OR TI = (detection) OR TI = (PCR) OR TI = (identification) OR TI = (method). Another search was performed using abstracts with the following strings: “AB = (*Candida auris*)” AND “AB = (molecular) AND AB = (diagnostic) OR AB = (diagnosis) OR AB = (screening) OR AB = (detection) OR AB = (PCR) OR AB = (identification) OR AB = (method)”.

### 2.3. Inclusion Criteria

Research articles were included if they fulfilled the following criteria:

(1) Original research study involving molecular diagnostics of *C. auris*;
(2) Studies involving DNA-based methodologies;
(3) Either prospective or retrospective laboratory studies;
(4) Studies published in the period between 1 January 2020 and 20 November 2024.

### 2.4. Exclusion Criteria

The exclusion criteria were as follows:

(1) Non-human laboratory data;
(2) Diagnostic methods other than molecular technologies (i.e., culture-based diagnostics);
(3) Diagnostic and typing methods such as WGS, multilocus sequence typing (MLST), amplified fragment length polymorphism (AFLP), and pulsed-field gel electrophoresis (PFGE);
(4) Studies other than original research (i.e., review articles);
(5) Manuscripts written in languages other than English;
(6) Studies published outside the defined study period.

### 2.5. Selection of Studies

Manuscripts obtained from each of the databases were incorporated into the EndNote X9 reference manager. After removing duplicate manuscripts, the title and abstract of the remaining were screened independently according to our defined inclusion criteria by two reviewers (R.C.-W.W. and A.L.-H.L.). After initial screening, full-text screenings were assessed for eligibility by two reviewers (R.C.-W.W. and A.L.-H.L.) to minimize the risk of bias. The reasons for excluding studies were documented during the screening. If there are any disagreements between two reviewers, a third reviewer (C.K.-C.L.) was responsible for the final decision. Relevant articles identified from the reference list of the included studies were added. To ensure the transparency in our selection process, a PRISMA flow diagram was generated to outline the process in detail.

### 2.6. Statistical Analysis

Although the findings in this review were mainly produced through narrative synthesis, a quantitative analysis was conducted where applicable. To compare the diagnostic value of various molecular assays, sensitivity and specificity were compared if such data were provided by the selected studies.

## 3. Results

### 3.1. Study Selection and Identification of the Included Studies

**Figure 1:**
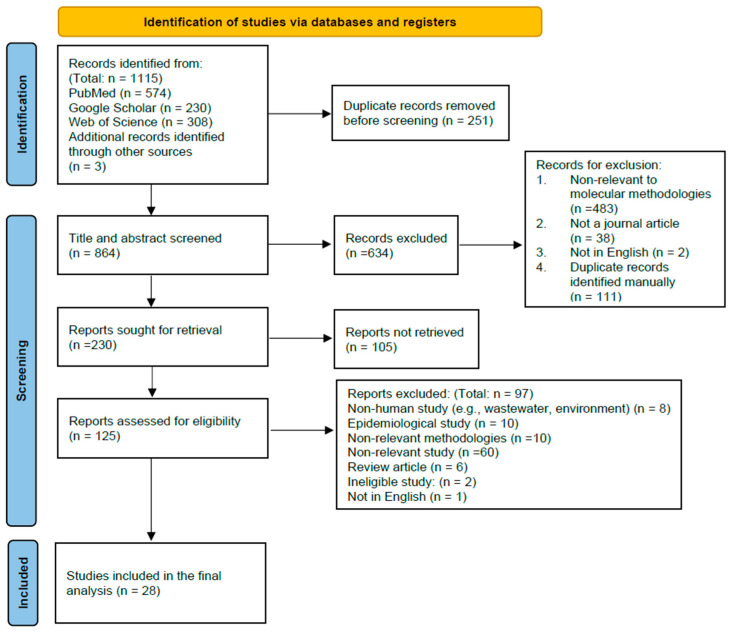
This PRISMA flow diagram illustrates the search process in this systematic review.

Figure 1 outlines the search process in this systematic review. Our literature search identified 1115 records. Before screening, 251 duplicate records were removed, leaving 864 records for the screening of title and abstract. During screening, 634 non-relevant records were excluded for not fulfilling the criteria, resulting in 230 reports sought for retrieval. Of these, 105 reports could not be retrieved, leaving 125 reports for eligibility assessment and undergoing full-text screening. At the final stage, 97 reports were excluded, leaving the 28 remaining studies included in the final analysis. The analyses were thematically grouped and summarized in three sections, including (i) sample pre-extraction procedures and nucleic acid extraction; (ii) commercial molecular assays; and (iii) laboratory-developed molecular assays.

To further enrich this review, we have supplemented our current practice in the molecular detection of *C. auris*, including sample pre-treatment, nucleic acid extraction methods, and real-time PCR by commercial assay (i.e., OLM AurisID (OLM, Newcastle Upon Tyne, UK)), with running LDT assays on high-throughput analyzers (i.e., BD MAX system (BD Diagnostics, Sparks, MD, USA)) and cobas Roche 6800 (Roche Diagnostics, Indianapolis, IN, USA).

### 3.2. Sample Pre-Extraction Procedures and Nucleic Acid Extraction

Among the 28 included studies, 19 studies described their sample pre-treatment and nucleic acid extraction procedures. These include 24 protocols supplemented with 3 protocols described by our group and are summarized in Table 1. Of these 27 protocols, manual extraction was the most common method used in the extraction of *C. auris* genomic DNA (74.1%, 20 out of 27), followed by automated extraction (22.2%, 6 out of 27) and semi-automated extraction (3.7%, 1 out of 27). These 27 protocols worked with various principles, including mixed-mode (e.g., column-based and bead-beating, column-based and mechanical-based, etc.) (9), mechanical-based (6), column-based (5), thermal-based (3), magnetic-based (2), and chemical-based (2) protocols.

Since *C. auris* has a thick fungal cell wall and is uniquely composed of mannans, chitin, and β-glucans, cell disruption pre-treatments such as magnetic, chemical, enzymatic, thermal, or mechanical-based steps are required to release its nucleic acids effectively [23,35,44,45]. Sample pre-treatment and nucleic acid extraction are still the prerequisite steps in most of the included studies, except the two studies performed by Bayona et al. and Ramrez et al. [10,46].

**Table 1:** Sample pre-extraction procedures and nucleic acid extraction.

Extraction Methods	Pre-Extraction Procedures	Extraction Kits Used	Equipment Used	Protocols	References
Column-based	/	Qiagen QIAamp kit, Qiagen QIAsymphony tissue kit (Qiagen, Hilden, Germany)	/	Manual extraction according to the manufacturer’s instructions.	[35]
Column-based	/	Qiagen Dneasy PowerLyzer microbial kit (Qiagen, Hilden, Germany)	/	Manual extraction according to the manufacturer’s instructions with the following modifications: (1) centrifuge the sample for 5 min; (2) incubate in elution buffer for 5 min; (3) elute the final sample in 50 μL.	[33]
Column-based	/	Qiagen kit (Qiagen, Hilden, Germany)	Qiagen BioRobot EZ1 (Qiagen, Tokyo, Japan)	Automated method according to the manufacturer’s instructions.	[13]
Column-based	Swabs were pooled in 1 mL water and vortex for 5 s	Qiagen virus mini kit (Qiagen, Hilden, Germany)	Qiagen BioRobot EZ1	Automated method according to the manufacturer’s instructions.	[14]
Column-based	/	Qiagen DNeasy UltraClean Microbial kit (Qiagen, Hilden, Germany)	/	Manual extraction according to the manufacturer’s instructions.	[23]
Column-based and mechanical-based	Homogenize the sample prior to DNA extraction	FastDNA kit (MP Biomedicals, Irvine, CA, USA), QIAamp DNA mini kit (Qiagen, Hilden, Germany)	MP Biomedical FastPrep homogenizer (MP Biomedicals, Irvine, CA, USA)	After homogenization, lysed sample will proceed with nucleic acid extraction using the QIAamp DNA mini kit.	[31]
Extraction methods	Pre-extraction procedures	Extraction kits used	Equipment used	Protocols	References
Column-based and bead-beating	/	Zymo Quick-DNA Fungal/Bacterial Miniprep kit (Zymo Research, Irvine, CA, USA)	Vortex Genie 2 (Scientific Industries, Bohemia, NY, USA)	Manual extraction for isolates: (1) inoculate the colonies in a ZR BashingBead lysis tube with 750 μL of BashingBead buffer; (2) vortex at maximum speed for 10 min with a Vortex Genie 2; (3) spin it down and filter the supernatent through the Zymo-Spin filter through centrifugation; (4) mix the filtrate with genomic lysis buffer and then transfer to a column; (5) final elution volume is 100 μL.	[29]
Column-based and mechanical-based	Add swabs to 1 mL MP Biomedical Lysing Matrix A tube. Then, homogenize the sample for 60 s under the speed of 6 m/s prior to DNA extraction	Qiagen QIAamp DNA Mini Kit (Qiagen, Hilden, Germany)	MP Biomedical FastPrep homogenizer	Semi-automated method: (1) quick spin the homogenized sample and pipette 200 μL into a 2 mL tube containing 20 μL of proteinase K; (2) mix 200 μL Buffer AL with 5 μL internal extraction control; (3) vortex the mixture for 15 s; (4) incubate at 56 °C for 10 min; (5) load the sample tube into the QIAcube and choose the “Body fluid spin protocol”; (6) extraction performed on the QIAcube; (7) final elution volume is 100 μL; (8) 6 μL DNA template for OLM AurisID real-time PCR assay.	This report
Chemical-based and thermal-based	/	MagCore Genomic DNA Whole Blood Kit (RBC Bioscience Corp., New Taipei City, Taiwan)	MagCore Plus II extractor (RBC Bioscience Corp., New Taipei City, Taiwan)	Manual extraction method includes cell lysis and protein degradation by heat-based lysis together with proteinase K and guanidine hydrochloride.	[9]
Magetic-based	/	Roche MagNA Pure 96 DNA and viral NA small volumn kit (Roche Diagnostics, North Ryde, NSW, Australia)	Roche MagNA Pure 96 (Roche Diagnostics, North Ryde, NSW, Australia)	(1) Add 4 mg/mL amplification control into 200 μL of sample; (2) DNA extracted by automated method and elute in final volume of 100 μL.	[47]
Magetic-based	/	/	easyMAG (bioMérieux, Durham, NC, USA)	Automated method according to the manufacturer’s instructions.	[25]
Extraction methods	Pre-extraction procedures	Extraction kits used	Equipment used	Protocols	References
Chemical-based	/	/	/	Manual extraction: (1) inoculate the swabs into 500 μL of Tris-EDTA (TE) pH 8.0 buffer; (2) for 50 μL of sample-containing TE buffer, add 5 μL of DiaSorin fungal lysis solution; (3) incubate the mixture at 60 °C for 30 min on a rocking incubator with 25 strokes per minute.	[26]
Chemical-based and thermal-based	Swab inoculate in wash solution	Kaneka DNA Extraction Kit 2 (Kaneka Co., Tokyo, Japan)	/	Manual extraction: (1) centrifuge 100 μL of sample at 13,000 r.p.m. for 10 min; (2) discard the supernatent and add 100 μL of Solution A to the pellets and vortex; (3) incubate at 98 °C for 8 min; (4) add 14 μL of Solution B and mix well; (5) 10 μL of DNA extract is used for LAMP reaction.	[15]
Thermal-based	/	Eazyplex LAMP kit (AmplexDiagnostics GmbH, Gars-Bahnhof, Germany)	/	(1) Addition of 25 μL of sample into the RALF buffer solution; (2) incubate at 99 °C for 5 min; (3) 25 μL of sample is used to test on the LAMP strip.	[27]
Mechanical-based	Cell suspension pre-wash by 500 μL of PBS	DM assay cartridge	OmniLyse device (Claremont BioSolutions, Upland, CA, USA)	Automated method based on the syringe-linked, bead-beating, and micro-motor-based principles; (1) 10 lysis cycles should be run for each of the samples; (2) mix 100 μL of lysate with 22 μL of magnetic bead buffer plus 1 μL of control DNA; (3) then, run it on a Point-of-Care handheld device with droplet magnetofluidics technology.	[29]
Mechanical-based	/	/	MP Biomedical FastPrep homogenizer	(1) Isolates were inoculated in 800 μL of solution (including 400 μL of TE buffer and 400 μL of phenol, chloroform, and isoamyl alcohol in a ratio of 25:24:1; (2) perform micro-bead shearing using the FastPrep homogenizer for 30 s under the speed of 6 m/s); (3) centrifuge the homogenate at 14,000 r.p.m. for 10 min; (4) 300 μL of the supernatent is then mix with 30 μL of 3 M sodium acetate and 900 μL of 100% ethanol; (5) cool the mixture at −20 °C for 30 min and centrifuge again at 14,000 r.p.m. for 10 min at 4 °C; (6) wash the pellet two times with 500 μL of 70% ethanol; (7) dry at 37 °C and dissolve the DNA in nuclease-free water.	[5]
Extraction methods	Pre-extraction procedures	Extraction kits used	Equipment used	Protocols	References
Mechanical-based	/	/	Precellys 24 homogenizer (Bertin Instruments, Montigny-le-Bretonneux, France)	Manual washing, freeze, thaw, and homogenization. (1) Centrifuge 200 μL of sample for 10 min at 15000 r.p.m. and remove the supernatant; (2) re-suspend the pellet in 100 μL of 1% PBS-BSA buffer; (3) add glass beads into the suspension; (4) freeze the suspension at −80 °C for 30 min and then incubate it on a heat block/water bath at 70 °C for 30 min; (5) homogenize the suspension for 20 s two times with 10 s intervals; (6) centrifuge the suspension for 10 min at 15,000 r.p.m; (7) then, proceed with PCR amplification for the supernatant.	[35]
Mechanical-based	Add swabs to 1 mL MP Biomedical Lysing Matrix A tube. Then, homogenize the sample for 60 s under the speed of 6 m/s before loading on the BD MAX	/	MP Biomedical FastPrep homogenizer	Automated method: (1) pipette 200 μL of the pre-treated specimen into a BD MAX ExK DNA-2 kit Sample Buffer Tube (SBT); (2) recap the inoculated SBT using a blue septum cap; (3) load the SBT on the BD MAX analyzer.	This report
Mechanical-based	Sample undergo ultrasonication before loading on the cobas Roche 6800	/	TS5 tube ultrasonicator (Rinco Ultrasonics, Romanshorn, Switzerland)	Automated method: (1) load 5 sample tubes at one time into the ultrasonicator with the following settings: sonication time: 25 s, pause time: 5 s, 10 cycles; force: 600 N; sonication amplitude: 40%; cycle energy limits: min = 200 J; max = 6000 J; program energy limits: min = 4000 J; max = 30,000 J; (2) load the processed samples on the cobas Roche 6800.	This report
Extraction methods	Pre-extraction procedures	Extraction kits used	Equipment used	Protocols	References
Mechanical-based and thermal-based	/	MagNA Pure LC Total Nucleic Acid Isolation Kit (Roche Diagnostics, Indianapolis, IN, USA)	Disruptor Genie (Scientific Industries Inc., Bohemia, NY, USA), MagNA Pure LC 2.0 (Roche Diagnostics, Indianapolis, IN, USA)	Isolates: (1) inoculate 1 μL loopful of colonies in 500 μL of water with silica glass or zirconia beads; (2) heat the sample at 95 °C for 10 min; (3) mechanical lysis by the Disruptor Genie for 2 min; (4) 5 μL of processed sample is use for PCR. Surveillance swabs: (1) place the swabs or inoculate 60 μL of the liquid in 600 μL of Tris-EDTA neutralization buffer; (2) shake with a thermomixer at 14,000 r.p.m. for 6 min at 100 °C. Whole blood: (1) 200 μL of sample is used for extraction on the MagNA Pure, with a final elution volume of 100 μL. Urine: (1) centrifuge 5 mL of urine; (2) 250 μL of the sample is heated at 95 °C for 5 min; (3) 200 μL of the processed sample is used for extraction on the MagNA Pure, with a final elution volume of 100 μL.	[12]
Chemical-based and thermal-based	/	/	/	(1) Suspend the yeast colonies in 20 μL of 20 mM NaOH; (2) incubate the suspension at 95 °C for 15 min; (3) 3 μL of lysed suspension is used for each of the 25 μL PCR reactions.	[20]
Thermal-based, bead-based, and chemical-based	/	/	Precelleys homogenizer	Colonies: (1) Colonies suspended in 50 μL of water; (2) boil at 95 °C for 20 min; (3) centrifuge at 5000 r.p.m. for 5 min; (4) supernatent then used as the PCR template. Manual extraction of clinical specimens using the glass bead–phenol–chloroform DNA extraction method. (1) Sample transfer to a tube containing glass beads and 400 μL of lysis buffer, 300 μL of phenol–chloroform; (2) homogenize the sample at 6000 r.p.m. for 3 × 60 s; (3) centrifuge the lysed sample at 5000 r.p.m. for 5 min; (4) transfer the supernatant to 300 μL of phenol–chloroform, and centrifuge again at 5000 r.p.m. for 5 min; (5) transfer the supernatant to a new tube, add an equal volume of chloroform, and centrifuge again at 5000 r.p.m. for 5 min; (6) transfer the supernatant to a new tube and add 2.5 and 0.1 in volume of absolute ethanol and 3 M sodium acetate, respectively; (7) freeze at −20 °C for 1 h, and centrifuge at 12,000 r.p.m. for 10 min; (8) wash the pellet in 70% cold ethanol, and air dry; (9) dissolve the sample in 30 μL of water for downstream PCR.	[39,48]
Extraction methods	Pre-extraction procedures	Extraction kits used	Equipment used	Protocols	References
Thermal-based	/	/	/	Yeast colonies: (1) suspend the colonies in 100 μL of water; (2) boil at 100 °C for 10 min; (3) centrifuge at 2000 g for 3 min; (4) supernatent then used as PCR template; (5) check DNA purity at A260/A280 using NanoDrop with a ratio of 1.7 to 2.1; (6) 100 pg to 1 μg of genomic DNA is used for PCR amplification..	[34]
Column-based and bead-based	/	Zymo Quick-DNA Fungal/Bacterial Miniprep kit	Vortex Genie 2	Manual extraction for isolates: (1) colonies suspended in PBS to yield a concentration of 1 × 10^5^ CFU/mL; (2) 200 μL of the sample is vortexed at maximum speed for 10 min with a Vortex Genie 2; (3) spin it down, and filter the supernatent through the Zymo-Spin filter through centrifugation; (4) mix the filtrate with a genomic lysis buffer and then transfer it to a column; (5) final elution volume is 35 μL.	[21]
Chemical-based	250 μL of sample (1 × 10^5^ CFU/mL) mix with 250 μL of binding buffer (4 M guanidine thiocyanate, 0.055 M Tris-HCL pH 7.5, 0.025 M pH 8 EDTA) and 10 μL of silica-coated magnetic beads	/	/	(1) Allow the samples to bind with the binding buffer for 10 min at room temperature; (2) pellet the silica-coated magnetic beads using a handheld magnet; (3) invert the tube to remove the solution; (4) resuspend the beads in 50 μL of wash solution with 0.05% Tween 20; (5) pellet the beads again, and remove the wash solution; (6) repeat for two times; (7) elute the beads in 10 μL of PCR buffer at 60 °C for 5 min.	[21]
Extraction methods	Pre-extraction procedures	Extraction kits used	Equipment used	Protocols	References
Thermal-based	Same as above	/	/	(1) Pre-heat 250 μL of the sample at 70 °C for 10 min before adding binding buffer and magnetic beads; (2) incubate for another 10 min; (3) same procedures as step 2 to 7 of the chemical-based protocol.	[21]
Mechanical-based	Same as above	/	/	(1) Inoculate 250 μL of the sample into 250 μL of pre-filled zirconia/silica beads, (2) vortex at maximum speed for 10 min with a Vortex Genie 2 before adding binding buffer and magnetic beads; (3) incubate for another 10 min; (4) combine with 250 μL of binding buffer and 10 μL of silica-coated magnetic beads; (5) same procedures as step 2 to 7 of the chemical-based protocol.	[21]

Qiagen column-based extraction kits were the most common method used in the included studies. Special models of vortex, such as Vortex Genie 2 with a special adapter, was used in multiple studies. Some of the studies used manual washing, or freeze and thaw pre-extraction steps, while mechanical-based methods are commonly used to disrupt the fungal cells [35]. Homogenizers, disrupters, and ultrasonicators were applied in cell disruption and lysis, including the MP Biomedical FastPrep homogenizer, Precellys 24 homogenizer, RINCO TS5 tube ultrasonicator, and Disruptor Genie. Various types of beads were used for bead-beating/mechanical-beating, including glass-beads, ZR BashingBeads, silica-beads, zirconia beads, and MP Biomedical Lysing Matrix A (ceramic sphere and garnet-beads).

### 3.3. Commercial Molecular Assays

Among the 28 included studies, 8 commercial molecular assays were verified, among which 6 were real-time PCR assays and 2 were LAMP assays (Table 2). A total of 1532 samples were tested on these eight commercial assays. Of these eight commercial assays, two of them were FDA-cleared (the Cobas ePlex BCID-FP panel and the BioFire FilmArray BCID2). Both multiplex PCR panels were verified with positive blood culture samples and were being run on a sample-to-answer platform. DiaSorin Molecular *C. auris* Simplexa assay was granted for the FDA De Novo in July 2024 and verified with the composite axilla and groin swabs in three included studies. Three assays obtained the CE-IVD mark, including the OLM AurisID assay, RealCycler *Candida auris* PCR, and Eazyplex Candida auris. The OLM AurisID assay was being verified by two studies and also our group, while nucleic acid extraction was omitted in the study by Bayona et al. [46].

For those studies where the results of sensitivity and specificity were provided, the real-time PCR assays had sensitivity that ranged from 94.9% to 100% and specificity that ranged from 98.2% to 100%. For the LAMP assays (Eazyplex Candida auris assay and LAMPAuris assay), the sensitivity ranged from 66% to 86% and the specificity ranged from 96.8% to 100%. While the sensitivity of LAMP assays was lower, their TAT was also shorter than real-time PCR assays.

### 3.4. Laboratory-Developed Molecular Assays

Among the 28 included studies, 16 LDTs supplemented with 2 LDTs described by our group were validated. The results are summarized in Table 3. All but one were real-time PCR assays. The remaining one was a conventional PCR assay. One study evaluated a point-of-care testing (POCT) device (i.e., POC.auris) that employed droplet magnetofluidic technology. A total of 23,303 samples were tested on these 18 LDTs.

Of these 18 LDTs, various internal controls were applied, including human β-globin, human beta-actin, lambda phage, bicoid gene, PhHV1 glycoprotein B gene, and proprietary internal control/amplification control. They were used to check for the sample quality, extraction quality, PCR inhibition, and PCR process. Nevertheless, multiple assays included in this review did not include an internal control. As a good molecular practice, clinical laboratories are highly recommended to include such controls in their LDTs in the molecular detection of *C. auris*.

For those studies where the results of sensitivity and specificity were provided, the real-time PCR assays had sensitivity that ranged from 44% to 100% and specificity that ranged from 92% to 100%.

**Table 2:** Commercial molecular assays.

Name of Assays	Target/Method	Sample Types	No. of Samples	Approval	Real-Time PCR Machines/Analyzer	Performance	References
Cobas ePlex BCID-FP (GenMark Dx, (Roche Diagnostics, Indianapolis, IN, USA))	Multiplex PCR	Positive blood culture (contrived)	49	FDA-cleared	cobas ePlex system (Roche Diagnostics, Indianapolis, IN, USA)	Sensitivity: 100%; Specificity: 100%; LoD:/; Reference method: culture	[49]
BioFire Filmarray BCID2 (bioMérieux, Durham, NC, USA)	Multiplex PCR	Positive blood culture	152	FDA-cleared	Filmarray system (bioMérieux, Durham, NC, USA)	Sensitivity: N.A.; Specificity: 100%; LoD:/; Reference method: culture	[50]
DiaSorin Molecular *C. auris* Simplexa (DiaSorin, Cypress, CA, USA)	*ITS2*/Real-time PCR	Axilla/groin	282	FDA De Novo granted in 7/2024	DiaSorin LIAISON MDX (DiaSorin, Cypress, CA, USA)	Sensitivity: 100%; Specificity: 100%; LoD: 1–2 CFU/PCR reaction; Reference method: culture	[26]
DiaSorin Molecular *C. auris* Simplexa	*ITS2*/Real-time PCR	Axilla/groin	60	FDA De Novo granted in 7/2024	DiaSorin LIAISON MDX	Sensitivity: 100%; Specificity: 100%; LoD: 26 CFU/PCR reaction; Reference method: culture	[10]
DiaSorin Molecular *C. auris* Simplexa	*ITS2*/Real-time PCR	Axilla/groin	25	FDA De Novo granted in 7/2024	DiaSorin LIAISON MDX	Sensitivity: 95.6%; Specificity: 100%; LoD: 600 CFU/mL; Reference method: another real-time PCR assay	[37]
RealCycler *Candida auris* PCR (Progenie Molecular, Valencia, Spain)	Real-time PCR	Axilla/groin	392	CE-IVD	Bio-Rad CFX96 (Bio-Rad Laboratories, Hercules, CA, USA)	Sensitivity: N.A.; Specificity: N.A.; LoD: 1 copy/μL; Reference method: culture	[9]
Name of assays	Target/Method	Sample types	No. of samples	Approval	Real-time PCR machines/Analyzer	Performance	References
OLM AurisID (OLM, Newcastle Upon Tyne, UK)	28S rRNA/Real-time PCR	Pharyngeal or axillary-rectal	113	CE-IVD	Bio-Rad CFX96	Without prior DNA extraction; Sensitivity: 96.6%; Specificity: 100%; LoD: 1 CFU/PCR reaction (500 CFU/mL); Reference method: culture	[46]
OLM AurisID	28S rRNA/Real-time PCR	Isolates	29	CE-IVD	ABI 7500 (Applied Biosystems, Foster City, CA, USA)	Sensitivity: N.A.; Specificity: 100%; LoD: 1 Copy/PCR reaction; Reference method: culture	[23]
OLM AurisID	28S rRNA/Real-time PCR	Axilla/groin, Sample spiked with isolates	95	CE-IVD	Qiagen Rotor-Gene Q (Qiagen, Hilden, Germany)	Sensitivity: 94.9%; Specificity: 98.2%; LoD: 15 copies/PCR reaction; Reference method: culture	This report
Eazyplex *Candida auris* (AmplexDiagnostics GmbH, Gars-Bahnhof, Germany)	LAMP	Isolates/Pharyngeal, axillary-rectal	51/152	CE-IVD	Genie HT (OptiGene Limited, West Sussex, UK)	Initial results without resolving the discrepancy: Sensitivity: 76–76.9%; Specificity: 96.8–100%; LoD: 1 CFU/LAMP reaction; Reference method: culture	[27]
Fungiplex Candida Auris Real-Time PCR (Bruker, Bremen, Germany)	Mating locus alpha	Isolates	29	RUO	ABI 7500	Sensitivity: N.A.; Specificity: 100%; LoD: 9 Copies/PCR reaction; Reference method: culture	[23]
Name of assays	Target/Method	Sample types	No. of samples	Approval	Real-time PCR machines/Analyzer	Performance	References
LAMPAuris	LAMP	Axilla/groin	103	/	Kaneka MyAbscope (Portable device) (Kaneka Co., Tokyo, Japan)	Sensitivity: 66–86%; Specificity: 97–100%; LoD: 20 copies/LAMP reaction; Reference method: culture and qPCR	[15]

Note: LAMP: loop-mediated isothermal amplification; LoD: limit of detection; N.A.: not applicable/information not provided in the original article.

**Table 3:** Laboratory-developed molecular assays.

Type of Methods	Target	Sample Types	No. of Samples	Internal Control(s)	Real-time PCR Machines	Performance	References
Real-time PCR	*ITS2*	Nares, axilla/groin	1414	Human β-globin gene	QuantStudio 6 Flex (Thermo Fisher Scientific Inc., Singapore)	Sensitivity: 100%; Specificity: 100%; LoD: 1 CFU/PCR reaction; Reference method: culture	[35,51]
Conventional /Real-time PCR	*ITS*	Isolates/clinical samples *	439/590	/	LightCycler 96 (Roche Diagnostics, Mannheim, Germany)	Sensitivity: N.A.; Specificity: N.A.; Conventional PCR: 100 CFU/PCR reaction; Real-time PCR: LoD: 10 CFU/PCR raction; Reference method: N.A.	[39]
Real-time PCR	*ITS2*	Isolates	104	/	ABI 7500	Sensitivity: N.A.; Specificity: 100%; LoD: 1 CFU/mL; Reference method: N.A.	[34]
Real-time PCR	*ITS2*	Sample spiked with isolates/groin, axilla	15/106	Amplificaton control	Roche LightCycler 480 (Roche Diagnostics, Mannheim, Germany)	Sensitivity: N.A.; Specificity: 100%; LoD: 1 CFU/PCR reaction; Reference method: culture	[47]
Real-time PCR	*ITS2*	Isolates/whole blood/Urine/Swabs	32/30/30/90	PhHV1 glycoprotein B gene	Roche LightCycler 480	Sensitivity: 93.3–100%; Specificity: 100%; LoD: 4–37 CFU/PCR reaction; Reference method: contrived samples spiked with colonies	[12]
Real-time PCR (Point-of-care)	*ITS2*	Isolates	16	Bicoid gene	POC.auris Droplet magnetofluidic device	POC.auris assay: Sensitivity: N.A.; Specificity: 100%; LoD: 300 CFU/mL; Reference method: manual real-time PCR assay	[29]
Type of methods	Target	Sample types	No. of samples	Internal control(s)	Real-time PCR machines	Performance	References
Real-time PCR	*ITS1/ITS2*	Nares, throat, axilla/groin, Urine	10,818 (pooled swabs) 4792 (ruine)	Human beta-actin	QuantStudio 7 (Thermo Fisher Scientific Inc., Singapore)	Sensitivity: 100%; Specificity: 99.4%; LoD: 100 CFU/mL; Reference method: culture	[14]
Conventional PCR	*ITS*	Isolates	23	/	Labcycler Basic thermocycler (Bioké, Leiden, The Netherlands).	Clade-specific PCR for rapid identification of *C. auris* and differentiation of 5 different clades. Results were confirmed by Sanger sequencing.	[5]
Real-time PCR	*ITS2*	Skin swabs, Sample spiked with isolates	145	Proprietary internal control	GeneXpert (LDT-Automated) (Cepheid, Sunnyvale, CA, USA)	CaurisSurV cartridge; Sensitivity: 92 to 97.5%; Specificity: 100%; LoD: 10.5 to 14.8 CFU/mL; Reference method: BD MAX PCR assay	[4]
Real-time PCR	*ITS2*	Axilla/groin	56	Proprietary internal control	Hologic Panther Fusion (LDT-Automated) (Hologic, Marlborough, MA, USA)	Sensitivity: 100%; Specificity: 97.96%; LoD: 0.47 CFU/mL; Reference method: culture	[6]
Real-time PCR	*ITS2*	Nares, axilla/groin	110	Bicoid gene	BD Max open system (LDT-Automated) (BD Diagnostics, Sparks, MD, USA)	Optimum extraction temperature on BD MAX is 75 °C for 20 min; Sensitivity: 96%; Specificity: 92%; LoD: 1 CFU/PCR reaction; Reference method: manual real-time PCR assay	[36,51]
Real-time PCR	*ITS*	Axilla/groin, Sample spiked with isolates	2566	Proprietary internal control	BD Max open system (LDT-Automated)	Sensitivity: 94.6%; Specificity: 100%; LoD: 31.25 copies/PCR reaction; Reference method: culture	This report
Type of methods	Target	Sample types	No. of samples	Internal control(s)	Real-time PCR machines	Performance	References
Real-time PCR	5.8s rRNA, *ITS2*	Axilla/groin, Sample spiked with isolates	104	Proprietary internal control	Roche 6800 cobas omni utility channel (LDT-Automated) (Roche Diagnostics, Indianapolis, IN, USA)	Sensitivity: 97.4%; Specificity: 100%; LoD: 270 copies/mL; Reference method: another real-time PCR assay	This report
Real-time PCR	5.8S rRNA, *ITS2*, 28S rRNA	Anterior nares	70	Lambda phage	Bio-Rad CFX 96	Sensitivity: 100%; Specificity: 100%; LoD: 10 CFU/PCR reaction; Reference method: culture	[24,33,52]
Real-time PCR	GPI	Isolates, Stool	128 1053	N.A.	Bio-Rad CFX 96	Sensitivity: N.A.; Specificity: 100%; LoD: 13 CFU/PCR reaction; Reference method: N.A.	[13]
Real-time PCR	GPI	Isolates	155	/	ABI 7500	Sensitivity: N.A.; Specificity: N.A.; LoD: 5 CFU/PCR reaction; Reference method: N.A.	[20]
Real-time PCR	/	Axilla/groin	195	/	/	Sensitivity: 44%; Specificity: 99%; LoD: N.A.; Reference method: culture	[38]
Real-time PCR	CDC protocol	Sample spiked with isolates	222	Bicoid gene	Bio-Rad CFX 96	Isolates (100 CFU/mL): Sensitivity: 100%; Specificity: 100%; LoD: 27.11 CFU/mL; Reference method: sample spiked with colonies. Isolates (10 CFU/mL): Sensitivity: 82.1%; Specificity: 100%; LoD: 27.11 CFU/mL; Reference method: sample specimens spiked with colonies.	[25]

Note: * Clinical samples included ear aspirations, oral swabs, sputum, nail/skin/groin/axillary scrapings; GPI: glycosyl-phosphatidylinositol protein-encoding gene; LoD: limit of detection; N.A.: not applicable/information not provided in the original article.

## 4. Discussion

### 4.1. Sample Pre-Extraction Procedures and Nucleic Acid Extraction

When comparing different extraction methods, PCR with manual extraction using washing, freeze, and thaw together with homogenization obtained earlier Ct values (3 log difference) than that with commercial column-based extraction kits (QIAamp and QIAsymphony tissue) [35].

From the authors’ experience, pre-extraction processing using the beads together with a homogenizer can significantly improve the recovery of *C. auris* DNA for downstream applications. Before proceeding with the sample for DNA extraction by either QIAcube (Qiagen, Hilden, Germany) (on use with the OLM AurisID assay) or BD MAX, we inoculated the specimens into the MP Biomedicals Lysing Matrix A tube (with a single ¼ inch ceramic sphere and irregularly shaped garnet particles) with 1 mL of nuclease-free water. Then, the sample was homogenized using the FastPrep homogenizer (MP Biomedicals, Irvine, CA, USA) for 60 s under a speed of 6 m/s. We found that there is an improvement and yielded earlier Ct values with at least 1 log difference compared to the PCR without prior pre-treatment by homogenization (unpublished data).

Manual shaking for 10 s with glass beads demonstrated a 20-fold improvement in the LoD when compared to procedures without manual shaking in a study [21]. Mechanical lysis with bead-beating through vortexing yielded the highest recovery of *C. auris* DNA. However, it may introduce PCR inhibitors into the reaction. This can be minimized by using an appropriate bead-beating material such as glass beads and adding bovine serum albumin [21].

A specific type of sample collection transport medium may contain PCR inhibitors (i.e., Copan Liquid Amies elution swab (ESwab) [35]. Therefore, it is necessary for the laboratory to optimize its pre-extraction process and nucleic acid extraction method against their specimen collection swabs being used. Removal of the transport medium can be achieved by centrifugation; to minimize the PCR inhibition, the pellet is recommended to be re-suspended in 1% PBS-BSA buffer [35]. In our laboratory, dry PCR-dual swabs are used for collection without any transportation medium.

Storage conditions and DNA stability can also affect the downstream assay performance. For example, DNA may degrade during long-term storage and multiple cycles of freeze–thawing [15].

### 4.2. Commercial Molecular Assays and Laboratory-Developed Molecular Assays

Mass screening is often required during hospital outbreaks in order to contain the ongoing nosocomial transmission of *C. auris*. There is currently no high-throughput FDA-approved test for colonization swabs. To enhance the laboratory testing capacity and minimize the hands-on time of laboratory staff, there is a pressing demand to automate the detection process using an open access channel of the existing high-throughput or sample-to-answer molecular platforms (i.e., Hologic Fusion, BD MAX, Roche 6800, and GeneXpert) [4,36]. A LDT TaqMan probe-based real-time PCR assay developed by the Lima et al. group was formerly evaluated on the BD MAX platform. Based on their protocol together with our own pre-extraction procedures, we have tested 2566 specimens on the BD MAX and yielded a sensitivity of 94.6% and specificity of 100% using a culture method as the reference (Table 1 and Table 3) [53]. The implementation of the LDT assay on the automated platform can allow technicians to walk away and perform other laboratory tasks during testing, offering flexibility for this laboratory to run the assays during non-office hours.

Composite axilla and groin swabs were the most common type of specimen received for molecular testing in this review. It is also recommended by the Centers for Disease Control and Prevention [37]. This sample type is suitable for *C. auris* colonization screening [12,17]. A recent study has demonstrated that the addition of nares to composite axilla and groin has yielded superior detection of *C. auris* [17,54]. Other specimen types tested in the included studies were urine, throat, whole blood, pharyngeal, and axillary-rectal swabs.

Owing to the limited number of positive patient samples available for test validation, especially in non-endemic areas, many studies used contrived samples spiked with *C. auris* isolates to simulate clinical samples. However, verification using real clinical specimens where available is highly recommended to assess the impact of the patient’s endogenous microbiota and human DNA on the performance of the assay. DNA-based methodologies in the detection of *C. aruis* are low-cost and fast. Nevertheless, it is recommended to validate the LDT assay with sequencing and compare its performance against *C. auris* culture before clinical implementation.

It is difficult to compare the analytical sensitivity (LoD) among the included studies. There is no standardized unit for the molecular targets of *C. auris*. In the literature, different units are being used, such as *C. auris* in CFU/PCR reaction, copy/PCR reaction, CFU/mL, copy/μL, and CFU/LAMP reaction. It is necessary for diagnostic laboratories to verify their own commercial assays and LDTs and determine their LoD before implementation in clinical service.

In the selection of an appropriate DNA-based assay in the detection of *C. auris*, it should be both sensitive and specific. Most of the studies have verified the analytical specificity against a wide scope of pathogens to ensure no cross-reactivity against closely related *Candida* species (e.g., *C. duobushaemulonii*, *C. haemulonii*) and pathogens other than *C. auris*. It should be aware that for the OLM AurisID assay, a high organism load (≥5 × 10^5^ copies/PCR reaction) of *C. duobushaemulonii, C. haemulonii*, and *C. pseudohaemulonii* can give rise to false-positive results [23]. One LDT assay detected these three pathogens, while their melting peak temperatures can differentiate from that of *C. auris* [12].

Various genetic loci or regions were used as the gene target for laboratory diagnosis among the included studies, including the *ITS1*, *ITS2*, *GPI*, 5.8S, 18S, and 28S rDNA regions. The *ITS* region was predominantly used, especially the *ITS2*. It is a good molecular target because there are multiple copies of *ITS* in a single genome, improving the sensitivity of the assay. GPI has also been used in two included studies. It is considered more specific than the other gene targets [13,20]. Apart from these gene targets, NADH dehydrogenase subunit 5 (*nad5*) was used in a recent study [55].

Most of the studies used culture results as the reference method, and multiple studies have selected their samples for test verification based on the known culture results. Ideally, the best way to conduct the study would be by testing the same sample, running two methodologies (i.e., DNA-based method vs. culture) in parallel, and adopting the double-blind principle to minimize sampling bias. Two studies have identified a potential clinical application in POCT such as LAMPAuris and the portable droplet magnetofluidic device together with a handheld OmniLyse device [15,29].

Surprisingly, there was a limited number of studies using receiver operating characteristic (ROC) curves as the statistical tool in their result analysis to determine the diagnostic performance of the assay. The omission of an ROC curve analysis for the diagnostic accuracy represents a missed opportunity to thoroughly assess the robustness of the molecular assays. To determine the LoD of the assays, linearity checks, standard curves, or probit regressions were performed on ten-fold serial dilutions of reference strains in most of the studies.

Other technologies applied in the identification or bacterial typing of *C. auris*, such as the new *C. auris*-specific T2 assay using the magnetic resonance (MR) technology and Bruker IR Biotyper using the Fourier transform-infrared spectroscopy, were not covered by this review. The T2 assay runs on the fully automated T2DX system and has been evaluated on axilla/groin composite swabs [56]. Our review was limited by focusing on DNA-based methodologies while identification and molecular epidemiological studies by WGS were excluded. As DNA-based assays (i.e., real-time PCR), rather than WGS, are commonly used in clinical laboratories in *C. auris* detection, it justifies our article selection strategy. Moreover, readers should be aware of the inherent limitations of molecular diagnostics assays as they cannot differentiate between live and remnant *C. auris* cells. To date, yeast isolation by sample culture is still considered as the gold standard and routine practice, even if its TAT is much longer than DNA-based methodologies. Culture is recommended by the CDC using chromogenic agar together with salt/dulcitol enrichment broth [25,57]. In our experience, culture is still required in active surveillance, as patients with low fungal load may not be detected by DNA-based methods.

## 5. Conclusions

To date, there is no systematic review explicitly focusing on the up-to-date molecular diagnostics of *C. auris* to cover the entire process, including sample pre-extraction procedures, nucleic acid extraction, and DNA-based detection. To our knowledge, this is the first review covering a detailed summary of the sample pre-extraction process and nucleic acid extraction methods, including bead-beating, mechanical, chemical, thermal, and column-based protocols. Here, we described a useful summary to enlighten readers from clinical microbiology laboratories on the nucleic acid extraction protocols and performance of various molecular diagnostic assays used for the detection of *C. auris*.

## Data Availability

The authors declare that the new data performed by our group can be shared by the corresponding authors upon reasonable request.
